# Preliminary Comparison of Molecular Antioxidant and Inflammatory Mechanisms Determined in the Peripheral Blood Granulocytes of COVID-19 Patients

**DOI:** 10.3390/ijms241713574

**Published:** 2023-09-01

**Authors:** Elżbieta Skrzydlewska, Wojciech Łuczaj, Michał Biernacki, Piotr Wójcik, Iwona Jarocka-Karpowicz, Biserka Orehovec, Bruno Baršić, Marko Tarle, Marta Kmet, Ivica Lukšić, Zlatko Marušić, Georg Bauer, Neven Žarković

**Affiliations:** 1 Department of Analytical Chemistry, Medical University of Bialystok, 15-222 Bialystok, Poland; wojciech.luczaj@umb.edu.pl (W.Ł.); michal.biernacki@umb.edu.pl (M.B.); piotr.wojcik53@gmail.com (P.W.); iwona.jarocka-karpowicz@umb.edu.pl (I.J.-K.); 2 Clinical Hospital Dubrava, HR-10000 Zagreb, Croatia; biserka.orehovec@gmail.com (B.O.); barsicbruno@gmail.com (B.B.); tarlemarko1@gmail.com (M.T.); zmarta.km@gmail.com (M.K.); luksic.ivica@gmail.com (I.L.); 3 School of Medicine, University of Zagreb, HR-10000 Zagreb, Croatia; 4 Division of Pathology, Clinical Hospital Centre Zagreb, HR-10000 Zagreb, Croatia; marus-ic_zlatko@yahoo.com; 5 Institute of Virology, Medical Center–University of Freiburg, 79104 Freiburg, Germany; georg.bauer@uniklinik-freiburg.de; 6 Laboratory for Oxidative Stress (LabOS), Ruđer Bošković Institute, HR-10000 Zagreb, Croatia

**Keywords:** antioxidants, COVID-19, eicosanoids, G protein-coupled receptors, granulocytes, inflammation

## Abstract

The aim of this study was to evaluate selected parameters of redox signaling and inflammation in the granulocytes of COVID-19 patients who recovered and those who died. Upon admission, the patients did not differ in terms of any relevant clinical parameter apart from the percentage of granulocytes, which was 6% higher on average in those patients who died. Granulocytes were isolated from the blood of 15 healthy people and survivors and 15 patients who died within a week, and who were selected post hoc for analysis according to their matching gender and age. They differed only in the lethal outcome, which could not be predicted upon arrival at the hospital. The proteins level (respective ELISA), antioxidant activity (spectrophotometry), and lipid mediators (UPUPLC–MS) were measured in the peripheral blood granulocytes obtained via gradient centrifugation. The levels of Nrf2, HO-1, NFκB, and IL-6 were higher in the granulocytes of COVID-19 patients who died within a week, while the activity of cytoplasmic Cu,Zn-SOD and mitochondrial Mn-SOD and IL-2/IL-10 were lower in comparison to the levels observed in survivors. Furthermore, in the granulocytes of those patients who died, an increase in pro-inflammatory eicosanoids (PGE2 and TXB2), together with elevated cannabinoid receptors 1 and 2 (associated with a decrease in the anti-inflammatory 15d-PGJ2), were found. Hence, this study suggests that by triggering transcription factors, granulocytes activate inflammatory and redox signaling, leading to the production of pro-inflammatory eicosanoids while reducing cellular antioxidant capacity through SOD, thus expressing an altered response to COVID-19, which may result in the onset of systemic oxidative stress, ARDS, and the death of the patient.

## 1. Introduction

Since the beginning of the pandemic, knowledge concerning the pathophysiology of COVID-19 and the pathogenic mechanisms of SARS-CoV-2 infection has increased significantly, and many biomarkers have been proposed to monitor the progression of the disease [1]. In addition, new and more efficient therapeutic and diagnostic approaches have recently been proposed to deal with future pandemic waves driven by coronaviruses [2]. Recent studies have also confirmed the key role of the immune response to the presence of the SARS-CoV-2 virus in both the pathogenesis and clinical manifestation of COVID-19, as a severe course of COVID-19 is associated with a strong impairment of the immune system. COVID-19 infection, on the one hand, is accompanied by an increase in the level of pro-inflammatory cytokines and the number of neutrophils; but on the other hand, it also leads to lymphopenia, resulting in an increased ratio of neutrophils to lymphocytes, which is a hallmark of severe COVID-19 [3,4]. Among the leukocytes, as cells of the innate immune system, granulocytes (mostly neutrophils) are the first to be recruited to the site of infection. They also play a key role in shaping the early response to infection as well as mediating the innate immune system and the acquired response. However, if granulocytes are not properly regulated, the inflammatory functions of these cells programmed to kill pathogens can lead to tissue damage [5].

After entering the body, the virus is recognized by toll-like receptors (TLR 7/8) that are expressed in granulocytes, leading to their activation. Consequently, the innate response is activated by maintaining the activation of several inflammatory mediators. This is accompanied by an imbalance in the intracellular environment, mainly due to the overproduction of reactive oxygen species (ROS) generated by granulocytes to remove pathogens. In this context, the most important are neutrophil granulocytes, which, in the process of “oxidative burst”, produce significant numbers of free superoxide anion radicals and hydrogen peroxide, which are essential for the elimination of pathogens [6]. In addition, the activation of TLR 7/8 also stimulates the release of ROS outside of the inflammatory cells. ROS interact with the main biomolecules, including lipids, leading to their oxidative modifications, resulting in damaged cell membranes. Damage to erythrocyte cell membranes leads to the release of iron ions, which catalyze the production of superoxide anion radicals and hydrogen peroxide. This situation is conducive to the occurrence of systemic oxidative stress, observed in the course of COVID-19 [7]. In addition, the activation of granulocytes and monocytes/macrophages promotes the spread of oxygen burst in response to SARS-CoV-2 infection, inducing an excessive production of ROS. As a consequence, oxidative modifications of cellular and extracellular bioactive molecules occur, thus contributing to the exacerbation of severe disease and chronic inflammation [8]. It is well known that overproduction of ROS leads to redox imbalance, which promotes the activation of the NFκB pathway, resulting in increased expression of cytokines and immunoglobulin G and further dysfunction of granulocytes and lymphocytes [9]. Severe COVID-19 cases show an increase in inflammatory cytokines indicative of a “cytokine storm”, seen especially in cases of extremely severe disease, resulting in disseminated intra-vascular coagulation [10]. This can initiate viral sepsis and inflammation of the lungs, leading to ARDS, respiratory failure, shock, multiple organ failure, and death [11].

It should be emphasized that “cytokine storm” is not an event that is only typical in aggressive COVID-19; the active elements of both the intracellular and extracellular tissue components are bioactive lipids, which participate in the functioning of these systems, e.g., as basic components of biological membranes and a source of energy [12]; however, in pathophysiological processes, innate immune cells, including granulocytes and monocytes/macrophages, are recruited to the site of infection, and there, they increase the production of ROS, which increases the release of bioactive lipids [13]; thus, polyunsaturated fatty acids and their metabolites play a key role in the development of infections, including COVID-19 [14]. Considering the above, it is also believed that susceptibility to SARS-CoV-2 is strongly associated with pre-existing conditions characterized by metabolic dysregulation, including changes in lipid metabolism [15]. Consequently, a COVID-19 infection causes massive cell death, which, in turn, triggers an “eicosanoid storm” of bioactive endogenous lipid mediators involved in the various stages of infection [16,17].

Therefore, the aim of this study was to analyze changes in the level of pro-inflammatory parameters, including those related to antioxidant signaling and lipid mediators, in the granulocytes of COVID-19 patients who had identical status upon admission to the hospital although, within a week, some recovered, while the others died from the disease.

## 2. Results

### 2.1. Comparison of Laboratory Parameters of the Two Groups of COVID-19 Patients with Normal Values

The results of basic laboratory blood parameters determined for the blood of healthy people and COVID-19 patients are presented in Table 1, which shows that only the relative proportion of granulocytes to IL-6 showed an upward trend, including a significant increase in the number of patients who died as a result of the disease compared with the values in the group of patients who survived.

### 2.2. SARS-CoV-2 and the Antioxidant Response

**Figure 1:**
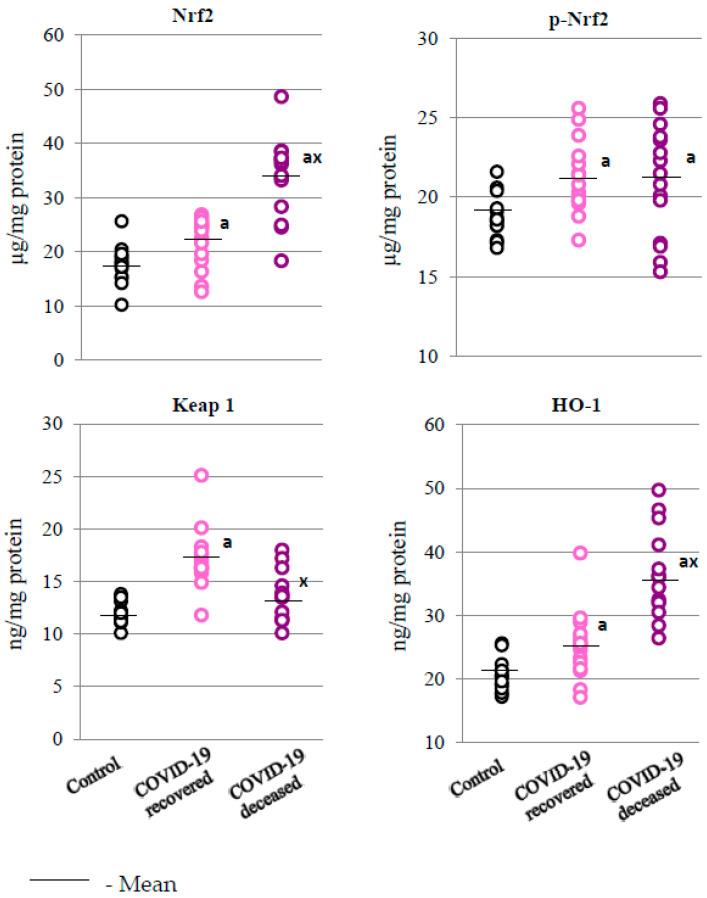
Levels of Nrf2, phosphorylated Nrf2 (p-Nrf2), and its inhibitor, i.e., Kelch-like ECH-associated protein 1 (Keap1), as well as heme oxygenase-1 (HO-1) observed in the granulocytes of recovered (n = 15) and deceased COVID-19 patients (n = 15), as well as persons from the control group (n = 15). The data points represent individual values per group: a—significantly different from control group, *p* < 0.05; x—significantly different from recovered COVID-19 patients, *p* < 0.05.

Changes in the antioxidant system of granulocytes taken form the peripheral blood of COVID-19 patients indicates an increased response to SARS-CoV-2 infection at the level of transcription factor Nrf2 and its phosphorylated form, as well as of the basic product of its transcriptional activity, i.e., heme oxygenase (HO-1), when compared to the values obtained for the control group (Figure 1). The level of the cytosolic Nrf2 inhibitor, i.e., the Keap1 protein, was also elevated in the group of patients who survived the disease, while it stayed at the control values in those patients who died, which corresponds to the direction of changes in the levels of Nrf2 and heme oxygenase.

**Figure 2:**
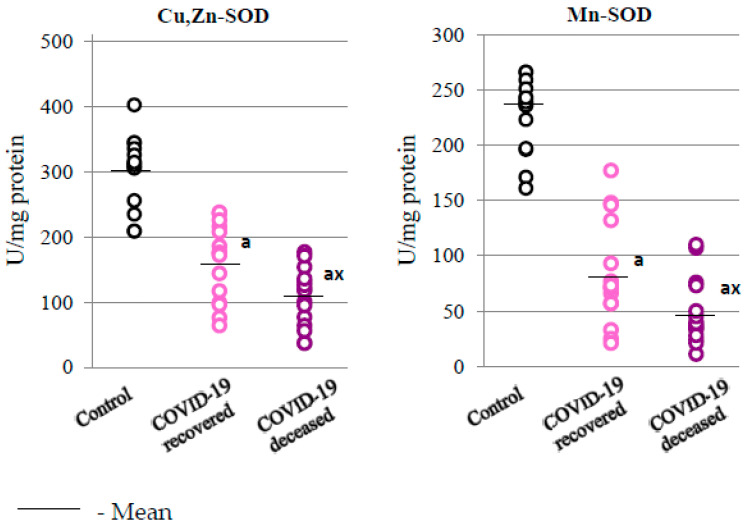
The activity of cytosolic superoxide dismutase (Cu,Zn-SOD) and mitochondrial superoxide dismutase (Mn-SOD) observed in the granulocytes obtained from recovered (n = 15) and deceased COVID-19 patients (n = 15), as well as from persons from the control group (n = 15). The data points represent individual values per group: a—significantly different from control group, *p* < 0.05; x—significantly different from recovered COVID-19 patients, *p* < 0.05.

At the same time, compared to the group of patients who survived, significant changes in the activity of basic antioxidant enzymes, i.e., superoxide dismutases—cytosolic (Cu,Zn-SOD) and mitochondrial (Mn-SOD)—responsible for dismutation of superoxide anions in various cell compartments, were observed in the granulocytes of patients who died from COVID-19 (Figure 2). Such varied responses at the level of the expression and activity of antioxidant proteins (HO-1 and SOD’s) may indicate modifications to the structure of antioxidant enzymes in disease conditions. 

### 2.3. SARS-CoV-2 and the Body’s Response to Inflammation

**Figure 3:**
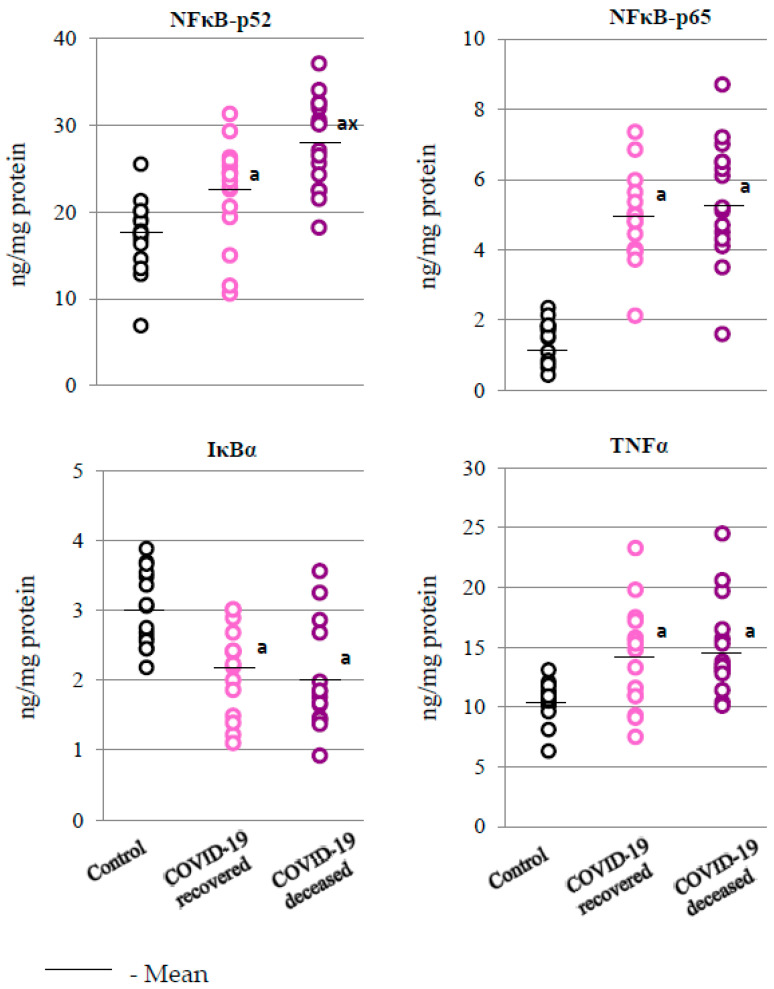
Levels of those proteins that play essential roles in the development of inflammation, such as two family members of nuclear factor kappa-light-chain-enhancer of activated B cells (NFκB p52 and NFκB p65), nuclear factor kappa-light-polypeptide-gene-enhancer of the B cells inhibitor, alpha gene (IκBα), and tumor necrosis factor alpha (TNFα) observed in the granulocytes obtained from recovered (n = 15) and deceased COVID-19 patients (n = 15), as well as from persons from the control group (n = 15). The data points represent the individual values per group: a—significantly different from control group, *p* < 0.05; x—significantly different from recovered COVID-19 patients, *p* < 0.05.

Since the transcription factor responsible for the antioxidant response (Nrf2) interacts with the basic transcription factor (NFκB) responsible for the inflammatory response, the results also show that SARS-CoV-2 infection is strongly associated with inflammation (Figure 3). This leads to disturbances in the metabolic pathway associated with the transcription factor NFκB. In the granulocytes of both groups of COVID-19 patients, increased expression of both NFκB subunits (p52 and p65) was observed. In addition, the level of p52 subunit in the granulocytes of deceased patients was significantly higher compared to the granulocytes of surviving patients. The increased level of NFκB was accompanied by a decrease in the level of the NFκB inhibitor, i.e., IκBα, which, in turn, could have contributed to the observed increase in the level of the product of NFκB transcriptional activity, i.e., TNFα, both in the group of patients who survived and in those who did not survive the infection (Figure 3).

**Figure 4:**
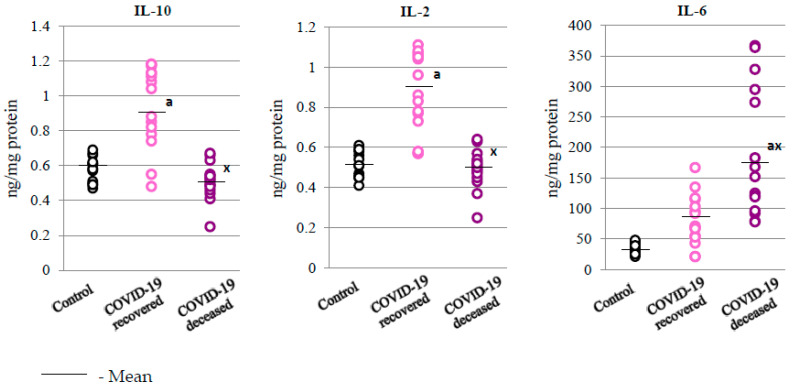
The level of anti-inflammatory interleukin 10 (IL-10) and pro-inflammatory interleukins, 2 (IL-2) and 6 (IL-6), observed in the granulocytes of recovered (n = 15) and deceased patients with COVID-19 (n = 15), as well as in those of people from the control group (n = 15). The data points represent the individual values per group: a—significantly different from control group, *p* < 0.05; x—significantly different from recovered COVID-19 patients, *p* < 0.05.

Further consequences of changes in the expression level of NFkB in the granulocytes of COVID-19 patients were changes in the expression of pro-inflammatory and anti-inflammatory interleukins; that is, in the granulocytes of patients who died from COVID-19, compared to COVID-19 survivors, there was an increase in the levels of IL-2 and IL-6 (proinflammatory interleukins) and a decrease in the level of the anti-inflammatory cytokine (IL-10) (Figure 4 and Table 1).

**Figure 5:**
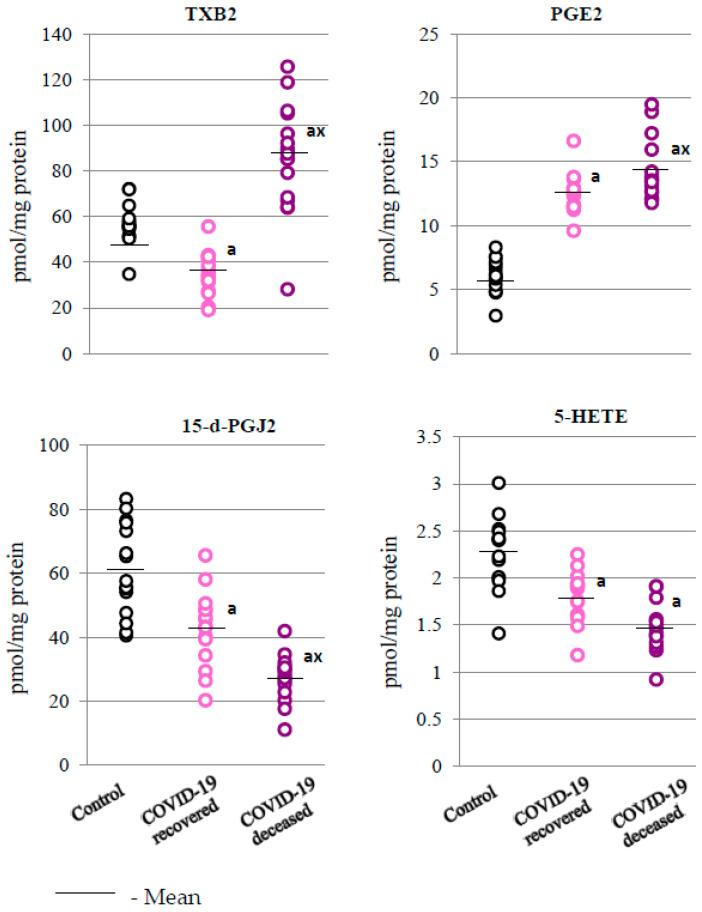
Levels of pro-inflammatory eicosanoids, thromboxane B2 (TXB2) and prostaglandin E2 (PGE2), and anti-inflammatory eicosanoids, 15-deoxy-delta12,14-prostaglandin J2 (15d-PGJ2) and 5-hydroxyeicosatetraenoic acid (5-HETE), observed in the granulocytes obtained from recovered (n = 15) and deceased COVID-19 patients (n = 15), as well as from persons from the control group (n = 15). The data points represent the individual values per group: a—significantly different from control group, *p* < 0.05; x—significantly different from recovered COVID-19 patients, *p* < 0.05.

The data obtained in the presented experiment also confirmed that as a result of SARS-CoV-2 infection, a differentiation in the level of lipid mediators occurred, especially concerning those involved in the regulation of processes related to inflammation, i.e., eicosanoids. In the granulocytes of patients who died from the disease, compared to recovered patients and to the control group, a significant increase in the levels of pro-inflammatory eicosanoids, namely, prostaglandin E2 (PGE2) and thromboxane B2 (TXB2), was observed (Figure 5). However, in the case of anti-inflammatory eicosanoids, 15-deoxy-delta12,14-prostaglandin J2 (15-d-PGJ2) and 5-hydroxyeicosatetraenoic acid (5-HETE), a decrease in their levels was observed, with statistically significant changes noted only for 15-d-PGJ2, in comparison to the control group and the group of patients who survived the infection.

**Figure 6:**
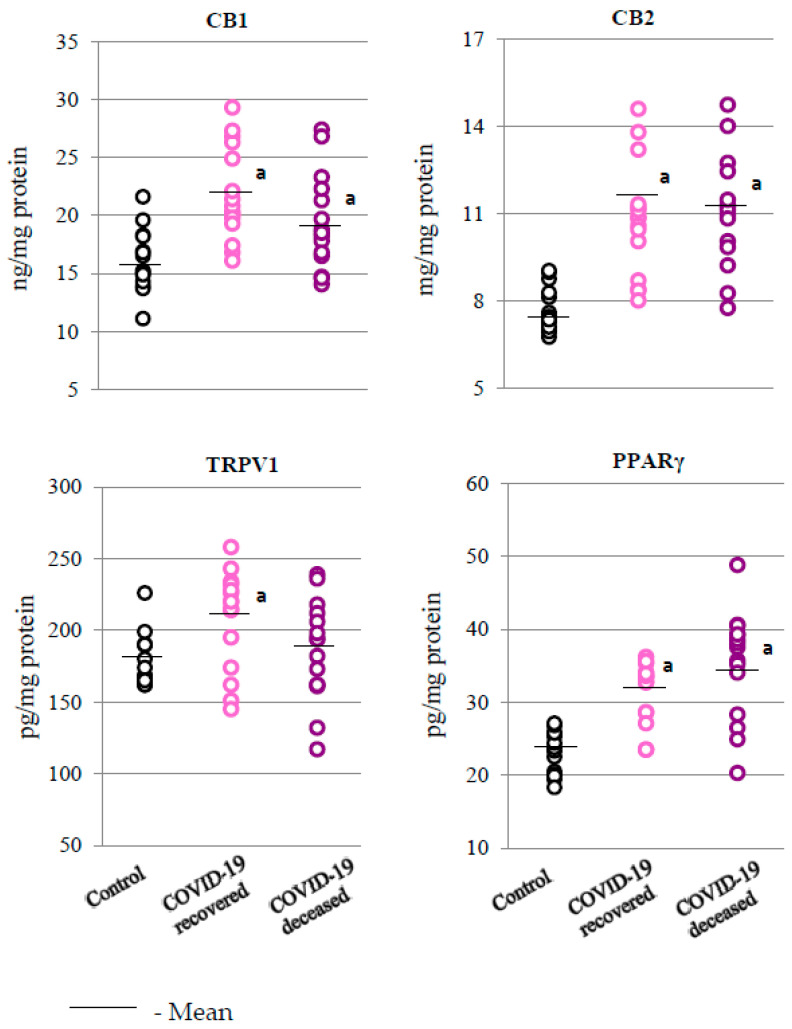
Levels of receptors involved in oxidative and inflammatory processes—cannabinoid receptors 1 and 2 (CB1 and CB2); transient receptor potential cation channel subfamily V member 1 (TRPV1); and peroxisome proliferator-activated receptor gamma (PPARγ)—observed in the granulocytes obtained from recovered (n = 15) and deceased COVID-19 patients (n = 15), as well as from persons from the control group (n = 15). The data points represent the individual values per group: a—significantly different from control group, *p* < 0.05; x—significantly different from recovered COVID-19 patients, *p* < 0.05.

The results obtained in the presented experiment also indicate that the redox balance and inflammatory process observed in the granulocytes of COVID-19 patients were also associated with changes in the activation of membrane receptors, especially those coupled with the G protein (Figure 6).

As the obtained results show, the expression of CB1/2 and PPARγ receptors was significantly elevated in the granulocytes of COVID-19 patients (both those who survived and those who died) compared to the control group. In contrast, a statistically significant increase in the expression of the TRPV1 receptor was observed only in patients who survived COVID-19.

## 3. Discussion

The immune response of the human organism to pathogens is a result of innate immunity, in which the main role is played by granulocytes, especially neutrophils, and adaptive immunity, whose effector cells are lymphocytes. Although the main granulocyte responses are associated with bacterial infections, they are also activated in viral infections [4], with the dominant activity of neutrophils in viral infections consisting of the degranulation of the granulocytes and the release of their toxic agents into the extracellular environment [18]. As a result, host cells/tissues are also damaged. This is intensified especially in the case of a significant increase in the number of neutrophils, as observed in sepsis [19]. The COVID-19 patients participating in the study were characterized by higher levels of neutrophils, particularly those who did not survive the infection, which is in line with the observations of other researchers [20].

In addition to their direct inflammatory effects, neutrophils also modulate the immune response through the release of cytokines, including IL-2, IL-6, and IL-10, determined in the current study. Interleukin 10 is believed to be the main anti-inflammatory cytokine; hence, its action may be one of the reasons explaining the better condition of some patients, assuming it does contribute to their recovery. Moreover, as a powerful negative regulator of COX-2 [21], IL-10 may decrease the production of prostaglandins, which are products of COX-2 action. On the other hand, IL-2 acts as an inducer of lymphocytes, further modulating inflammatory reactions and promoting immunosuppressive Th subpopulations, thus eventually suppressing inflammation [22]. Therefore, its higher levels, as observed in this study, may be partially responsible for the cessation of inflammatory processes leading to recovery in COVID-19 patients. On the contrary, proinflammatory cytokine IL-6 is believed to be a marker of poor prognosis in COVID-19, as has also been observed in a recent study [23].

Moreover, it is known that soluble immune complexes and afucosylated SARS-CoV-2 IgG are among the early triggers of immune pathology in patients with severe or critical course of COVID-19 [24]. Thus, stimulation of FcγIIIA receptors on neutrophils by SARS-CoV-2-specific soluble immune complexes and afucosylated IgG may be a link between early immune effects and later ROS-mediated pathogenic effects of activated granulocytes. Infection with SARS-CoV-2 is associated with increased generation of ROS, which promotes the intensification of oxidative conditions in granulocytes, notably their oxidative burst [4]. Therefore, the observed reaction of the granulocytes is the activation of nuclear erythroid factor 2 (Nrf2), which is responsible for the biosynthesis of cytoprotective proteins, including antioxidants [25]. Under physiological conditions, the Nrf2 inhibitor, i.e., the Keap1 protein, in cooperation with the E3 ubiquitin ligase, participates in the regulation of Nrf2 activity, directing it to ubiquitination and proteasomal degradation [26]. However, oxidative stress was observed in the plasma of COVID-19 patients with increased lipid peroxidation, whereas elevated levels of 4-hydroxynonenal (4-HNE) favor the modification of Keap1 sulfhydryl groups, which promotes dissociation of the Nrf2-Keap1 complex and the transcription of antioxidant protein genes [27]. The observed reduction in the level of free Keap1 in the granulocytes of patients who died from COVID-19 was associated with a significant increase in Nrf2 levels, which, in turn, promotes increased transcriptional activity of Nrf2 manifested in increased HO-1 levels. Similar results were obtained in other viral infections, including those caused by influenza and respiratory syncytial virus [28]. It is known, however, that heme oxygenase is involved in the degradation of heme to biliverdin, Fe^2+^, and CO, which are believed to be active against SARS-CoV-2 [29]. However, both CO and biliverdin are pro-inflammatory factors [29]; therefore, the observed activation of the Nrf2/HO-1 pathway, instead of alleviating the effects of the disease, may be associated with the death of patients due to an increased pro-inflammatory response.

Studies by other authors showed that overexpression of HO-1 in endothelial cells inhibits TNF-α-induced expression of pro-inflammatory adhesion molecules (E-selectin and VCAM-1), which occurs at the mRNA level by interfering with the rate of transcription [29]. This suggests that the Nrf2 pathway may interact with the NFκB pathway to inhibit its activation by upregulating HO-1, thereby reducing cytokine release. However, NFκB may also modify the expression of ARE-regulated genes, as the canonical subunit of NFκB-p65 antagonizes the formation of the Nrf2-ARE adduct, which reduces the transcription of ARE-dependent genes, including antioxidant proteins [30]. The potential participation of Nrf2 in the development of COVID-19 is also evidenced by the fact that Nrf2 activators, i.e., 4-octyl itaconate (4-OI) and dimethyl fumarate (DMF), inhibit inflammation in COVID-19 [31]. In addition, it is believed that Nrf2 induction may alleviate the dysfunction of regulatory T cells and consequently offer protection against autoimmune disorders [32], as confirmed by studies showing that patients with pre-existing susceptibility to autoimmune diseases are at a higher risk of severe COVID-19 [33]. Nevertheless, this study does not confirm the assumed positive role of Nrf2 activation in COVID-19 infection. This may be due to the fact that Nrf2 activation is associated with oxidative stress, which, in the case of granulocytes, is linked with oxidative burst. However, while the highest activation of Nrf2 is observed in the most severe cases, the effects of Nrf2 may not be strong enough to reduce the effects of an oxidative burst.

Genes regulated by Nrf2 also include those of superoxide dismutases (SOD1/2), i.e., enzymes that metabolize superoxide anion radicals to hydrogen peroxide [34]. In the granulocytes of patients who died as a result of COVID-19, a dramatic decrease in the activity of both isoforms of cellular superoxide dismutase have been observed when compared to patients who survived. This may suggest that despite the probably increased biosynthesis, as indirectly indicated by the increased expression of Nrf2 and HO-1, enzyme proteins were inactivated, presumably as a result of oxidative modifications, e.g., by electrophiles such as ROS, 4-HNE, or 15-d-PGJ2, which increase significantly during infection. However, taking into account literature data indicating the suppression of genes related to the Nrf2 antioxidant response in lung biopsies of COVID-19 patients and in in vitro experiments, which showed that the expression of Nrf2-dependent proteins is also reduced [31], it is likely that this pathway may be reduced in COVID-19 patients, or that the metabolic responses of the lung cells and granulocytes of COVID-19 patients may be different. The results of granulocyte SOD activity obtained in this study match those of reduced SOD activity observed in the plasma of COVID-19 patients [35]. In turn, the reverse response of granulocytes in the severe, lethal course of COVID-19 to HO-1 may be a specific gene response of this protein or depend on the type of cells in which the antioxidant response was analyzed. A similar response was observed in in vitro experiments on human alveolar cells infected with other respiratory viruses, such as influenza A virus (IAV H1N1), which was associated with the degradation of transcription factor 1 (Sp1), which is involved in the expression of the SOD1 gene [36].

It can therefore be assumed that the reduced activity of superoxide dismutases in patients who died from COVID-19 causes an increase in the concentration of superoxide anions and a decrease in the concentration of hydrogen peroxide generated as a result of SOD’s activity. On the other hand, free superoxide anions also show the ability to modulate myeloperoxidase (MPO), a key neutrophil enzyme, which affects the synthesis of HOCl [37]. Therefore, the reduced activity of SOD favors a reaction between superoxide anions and HOCl, causing the formation of hydroxyl radicals, and a reaction with nitric oxide, leading to the formation of peroxynitrites [38]. Both processes can have a strong impact on the efficiency of lipid peroxidation and the subsequent generation of active signaling molecules, including 4-HNE.

Disturbed antioxidant efficiency as a result of infection promotes increased generation of ROS, which may regulate the degradation of IκBα, which plays an important role in the activation of NFκB signaling pathways, causing the release of p50 and p65 dimers and their translocation to the nucleus for the transcription of inflammatory genes, including TNFα [39,40]. However, in COVID-19 survivors, the degradation of IκBα probably plays a minor role in the activation of NFκB signaling pathways; the results of this study showed only a small reduction in the level of this protein in the granulocytes. This may be related to the fact that the Nrf2 inhibitor complex, i.e., Keap1/Cul3, can direct IκBα to ubiquitination and degradation [41], and in patients who did not survive COVID-19, the level of Keap1 showed a downward trend.

One of the NFκB-IκB complex inhibitors, i.e., 4-HNE, is the main bioactive product of arachidonic acid peroxidation, the level of which increases oxidative stress under the influence of COVID-19 infection [41,42], which may be associated with systemic vascular stress in patients, especially those who died from COVID-19 [27,43]. It is known, however, that NFκB is necessary to maintain immune homeostasis and prevent autoimmunity caused by SARS-CoV-2 [39]. The increase in the level of the NFκB subunit, i.e., p52 protein, observed in this study in patients with the fatal form of COVID-19 may suggest that it is caused by oxidative stress and the expression of cytokines, including IL-6, which stimulates inflammatory processes. NFκB also influences the induction of the innate immune response, leading to the activation of cells involved in adaptive immunity, especially T lymphocytes, which leads to autoimmune inflammation and, consequently, to the release of inflammasome components [44].

Recent studies confirm that the strongest mediators of inflammation are metabolites of endogenous lipids, especially arachidonic acid, which are believed to be responsible for the body’s response to viral infection. Recent studies confirm that among the numerous compounds modulating the body’s immune response to viral infection, metabolites of endogenous lipids, mainly arachidonic acid, also play an important role [45,46]. In the presented study, increased levels of pro-inflammatory eicosanoids (PGE2 and TXB2) and decreased levels of anti-inflammatory eicosanoids (15-d-PGJ2) were observed in the granulocytes of patients who died from COVID-19. In addition, literature data indicate that significantly elevated levels of pro-inflammatory PGE2, TXB2, 12-HHTrE, and LTB4 were found in the BAL fluids of COVID-19 patients [47]. It is believed that elevated levels of PGE2 may be particularly responsible for the release of pro-inflammatory cytokines [48], which can be related to the elevated level of IL-2, as observed in this study in the granulocytes of patients who survived the disease, and of IL-6, as observed mainly in the granulocytes of patients who died as a result of COVID-19. In addition, it is known that PGE2 may aggravate intravascular thrombosis [49], which is another issue in patients with severe and fatal course of COVID-19. Elevated levels of TXB2 in patients who died as a result of COVID-19 indicate some similarity in the response observed in patients with pneumonia [50]. TXB2 is a potent activator of platelet aggregation whose effectiveness is enhanced by IL6, which is extremely elevated in patients who have died of the infection. Consequently, an elevated TXB2 level may indicate the presence of severe microthrombosis in these patients, which has previously been demonstrated in patients with COVID-19 [50,51].

The increase in the level of pro-inflammatory lipid mediators in the granulocytes of patients who did not survive COVID-19, as observed in this study, was also accompanied by a significant decrease in the level of anti-inflammatory prostaglandin 15-d-PGJ2, which inhibits COX2 activity, especially in inflammatory conditions [52]. In addition, 15d-PGJ2, as an electrophilic molecule, forms adducts with proteins, changing their structure and functions, which allows them, for example, to modify both the inflammatory process (NFκB pathway) [52] and the antioxidant response (via Nrf2 signaling) [53] in the granulocytes of patients who died from COVID-19, as observed in this study. It is known that PUFAs’ metabolites may also influence inflammation and oxidative states by stimulating membrane receptors, mainly PPARγ and CB1/2. This may be the case, in particular, with respect to PGE2 and TXB2, which are significantly elevated in patients with severe COVID-19. In addition, in patients with severe disease, a tendency to reduce the level of 5-HETE was observed, which may indicate a reduced activity of 5-lipoxygenase (5-LOX) as a result of the action of prostaglandin PGE2 [54]. On the other hand, activation of CB2 receptors by inhibiting leukocyte recruitment and reducing the synthesis of pro-inflammatory cytokines, as well as lipid and ROS mediators, promotes both anti-inflammatory and antioxidant effects [55,56]. This may be an important element of the altered response of the immune system to SARS-CoV-2 infection in terminally ill patients, which is associated with an altered lipid metabolism [57].

Finally, it should be noted that in this study, those patients who recovered and those who died from COVID-19 were age and gender matched. This is an important factor for the following reason: from the beginning of the pandemic [58] until the end of its six waves [59], disputes that concerned the higher risk of lethal outcome of a SARS-CoV-2 infection in elderly people, especially men, were common, while comorbidities and the variant of virus appeared to be more relevant predictive factors. However, the patients involved in this study had no comorbidities and were all hospitalized during the same wave of the pandemic in December 2020, so the obtained results do indeed reflect the differences associated with different outcomes of the severe disease itself.

## 4. Materials and Methods

### 4.1. Samples Collection

Granulocytes were isolated from the peripheral blood of patients admitted to the Dubrava Clinical Hospital in Zagreb during December 2020 in order to assess metabolic changes in COVID-19 patients. Patients included in the study have provided their signed informed consent. The study was approved by the Ethical Committee (2020-1012-13) of the Dubrava Clinical Hospital in Zagreb. Terminal patients or those with other chronic diseases such as cancer, an autoimmune disease, or diabetes were not enrolled in the study as this could affect the results. Furthermore, none of the patients had been treated with drugs that could affect lipid metabolism or oxidative stress (such as NSAIDs), and they were not on any specific diet prior to hospital admission. However, patients fasted due to the severity of the disease.

The patients were treated following the standard guidelines for COVID-19 therapy in addition to individual estimation of the severity of the disease of each patient based on the need for respiratory support to maintain capillary oxygen saturation above 93%. Severe disease was defined as a need for 8 L/min of supplemented oxygen during spontaneous breathing or for high-flow oxygenation or mechanical ventilation to achieve the defined target. The differences between the treatments of patients in accordance with personalized medicine guidelines did not influence the results of the study because the blood of patients was collected upon admission to the hospital, i.e., before any treatment was applied.

Out of the group of 80 initially included patients, blood samples of 15 patients who survived and of 15 patients who died from COVID-19 were selected for the study, with the aim of excluding unreliable samples (mostly due to insufficient blood volume), samples of blood collected from patients who passed away within a couple of hours after admission, or those who differed too much with respect to their age (below 40 or over 80 years of age). It should be emphasized that the clinical hospital in Dubrava served as the national COVID-19 center and received, on the one hand, patients with the most severe disease; while on the other, the hospital also had to receive patients whose symptoms were not as severe but who were received according to their residential address in the vicinity of the hospital. Hence, the maximum number of those who died within a week, but who did not differ upon admission from those who survived according to any relevant parameter, was 15. Eventually, 15 COVID-19 patients who survived (female/male: 7/8; average age: 57 (49–71)) and 15 patients who died from COVID-19 (female/male: 7/8; average age: 66 (60–73)) were enrolled. The maximum number of patients who died within a week of admission and whose clinical parameters assessed at the time of admission were not significantly different from those of the surviving patients was 15 (Table 1). The control group consisted of 15 healthy donors (female/male: 7/8; average age: 55 (47–58)). There was no difference in the body mass index between the patients and the healthy control subjects. 

The age differences between the COVID-19 patients and the healthy persons were the consequence of our inability to obtain blood samples from elderly, apparently healthy people during the peak of the pandemic and the lock-down regime. The results of basic blood laboratory parameters determined in the peripheral blood of healthy people and COVID-19 patients are presented in the Section 2.

To obtain the granulocyte fraction, blood samples (collected into EDTA tubes) from patients/healthy subjects were centrifuged in two stages. In the first step, the samples were centrifuged at 3000× *g* at 4 °C in order to separate the plasma, the buffy coat, and the erythrocytes. The obtained buffy coat was loaded onto Gradisol G (Aqua-MedZPAM—KOLASA, Łódź, Poland) and centrifuged at 300× *g* for 25 min at room temperature to acquire a homogeneous fraction of granulocytes. The granulocytes were then washed 3 times with PBS (centrifugation at 300× *g* for 3 min) and resuspended in PBS. The obtained cell fraction purity had to be at least 90% and was examined microscopically (Nikon Eclipse Ti, Nikon Instruments Inc., New York, NY, USA). For this purpose, a drop of cells was applied to the microscope glass and a manual smear was made. Due to the fact that the blood samples were collected at different times, different measurement conditions may have influenced the results. To avoid such a situation, microscope calibration was carried out before each measurement, i.e., after the blood samples were collected. For cell staining in randomly selected samples, the eosin methylene blue method was used. The high reproducibility of the purity of the samples allowed us to confirm the morphology of the remaining samples without staining. All the resulting fractions were immediately frozen and stored at −80 °C until analysis. All measurements were carried out in cell lysates, which were obtained by sonicating the cells on ice immediately after thawing, using a Ultrasonic Homogenizer Sonic Ruptor 400 (Omni-International Homogenizer Company, Kennesaw, GA, USA) and applying 7 1-second strokes at 50% of the power of the device in 3 repetitions for each sample.

### 4.2. Determination of Superoxide Dismutases Activity

Cytosolic superoxide dismutase (SOD) activity dependent on the copper and zinc ions (Cu,Zn–SOD–EC.1.15.1.1) of granulocytes was assayed using the spectrophotometric (480 nm) method described by Misra and Fridovich [60] and modified by Sykes [61]. One unit of SOD was defined as the amount of enzyme required to inhibit the auto-oxidation of epinephrine by 50%. The Cu,Zn–SOD activity in granulocytes was expressed as units per mg of protein.

Manganese-dependent superoxide dismutase (Mn-SOD–EC.1.15.1.1) activity was measured as described by Galler and Winge [62] in isolated granulocytes. All measurements were performed in the presence of 2 mM KCN to inhibit 90% of the activity of the Cu,Zn-SOD enzyme. One unit of SOD was defined as the amount of enzyme required to inhibit the auto-oxidation of epinephrine by 50%. The Mn-SOD activity in granulocytes was expressed as units per mg of protein.

### 4.3. Determination of Expression of the Analyzed Proteins

The immunosorbent assay (ELISA) was used for measuring protein expression in granulocytes) [63]. Lysates of granulocytes were incubated in ELISA plates (Nunc Immuno MaxiSorp, Thermo Scientific, Waltham, MA, USA) for 3 h in 4 °C with a blocking solution (5% fat-free dry milk in a carbonate binding buffer). Supernatants were washed with PBS containing 0.1% Tween 20; then, the samples were incubated overnight with the primary antibody at 4 °C (Nrf2, HO-1, TNFα, NFκB p52 (host: rabbit), Keap1, TRPV1 (host: mouse) (Sigma-Aldrich, St. Louis, MO, USA); CB1, CB2, NFκB p65, IL-10 (host: mouse) (Santa Cruz Biotechnology, CA, USA); p-Nrf2, PPARγ (host: rabbit), IL-2 (host: mouse) (Invitrogen, Waltham, MA, USA), IκBα (host: mouse) (Abcam, Cambridge, UK)). The samples were washed with PBS containing 0.1% Tween 20 and then incubated with a peroxidase blocking solution (3% hydrogen peroxide; 3% fat free dry milk in PBS) at room temperature for 30 min. After 1 h of incubation with a secondary antibody, i.e., goat anti-rabbit/mouse EnVision+ Dual Link/HRP solution (1:100) (Agilent Technologies, Santa Clara, CA, USA) at room temperature, the antibodies were removed and the samples were incubated for 40 min with a chromogen substrate solution (0.1 mg/mL TMB, 0.012% H_2_O_2_). A total of 2 M sulfuric acid was added to all the samples to stop the reaction. Absorbance was read at 450 nm within 10 min. The standard curve was plotted with the aim to calculate the concentration of each protein (NFκBp52 (LLQ = 0.5 ng/mL), TRPV1 (LLQ = 0.1 pg/mL), Lifespan Biosciences, Seattle, WA, USA; NFκBp65 (LLQ = 0.02 ng/mL), OriGene Technologies, Rockville, USA; TNFα (LLQ = 0.4 ng/mL), Merck, Darmstadt, Germany; PPARγ (LLQ = 1 pg/mL), IL-10 (LLQ = 0.015 ng/mL), IL-6 (LLQ = 0.2 ng/mL), IL-2 (LLQ = 0.015 ng/mL), Fine, Test Wuhan, Hubei, China; CB1 (LLQ = 0.2 ng/mL), IκBα (LLQ = 0.07 ng/mL), Abcam, Cambridge, UK; CB2 (LLQ = 0.2 ng/mL), Abnova, Taipei, Taiwan; Keap1 (LLQ = 0.5 ng/mL), Sino Biological, Beijing, China; HO-1 (LLQ = 0.6 ng/mL), Enzo Life, Farmingdale, NY, USA; Nrf2 (LLQ = 0.6 μg/mL), p-Nrf2 (LLQ = 1.0 μg/mL), MyBioSource, San Diego, CA, USA).

### 4.4. Determination of the Level of Eicosanoids

A total of 100 μL lysates of granulocytes were thawed on ice and spiked with 10 µL of internal standards solution (100 ng/mL TXB_2_-d_4_, PGD_2_-d_4_, 15-d-PGJ_2_-d_4_, and 15-HETE-d_8_), deproteinized with 500 μL acetone, vortexed, and loaded onto SPE cartridges (Oasis^®^ HLB 3 cm^3^ (60 mg) SPE cartridges (Waters, Milford, MA, USA)) for SPE extraction and purification. The obtained eluates were evaporated under a gentle stream of nitrogen at room temperature and reconstituted in ACN/H2O (8:2) with 0.1% acetic acid. The obtained samples were transferred into LC vials and subjected to LC–MS/MS analysis immediately. Chromatographic separation was carried out on Eclipse Plus C18 column (2.1 × 100 mm, 1.8 µm) in gradient mode with the use of mobile phases consisting of water with 0.1% acetic acid (mobile phase A) and acetonitrile (mobile phase B). The gradient elution was as follows: 0.0–1.0 min, 25 to 40% B; 1.0–2.5 min, 40 to 42% B; 2.5–4.5 min, 42 to 50% B; 4.5 to 10.5 min, 50 to 65% B; 10.5–12.5 min, 65 to 75% B; 12.5–14.0 min, 75 to 85% B; 14.0–14.5 min, 85 to 95% B; 14.5–15 min, 95 to 25% B; and 15.0–16.0 min, 25% B. All analyses were performed using a Shimadzu UPLC system (Nexera X2) coupled to an Shimadzu 8060 Triple Quadrupole mass spectrometer (Shimadzu, Kyoto, Japan) equipped with an electrospray ionization (ESI) source working in negative mode [64]. The precursors to the product ion transitions were as follows: *m*/*z* 351.3→271.2 for PGE_2_; *m*/*z* 315.2→271.2 for 15-d-PGJ_2_; *m*/*z* 369.3→169.1 for TXB_2_; *m*/*z* 319.2→257.2 for 5-HETE; *m*/*z* 355.0→275.3 for PGD_2_-d_4_; *m*/*z* 373.0→173.1 for TXB_2_-d_4_; *m*/*z* 319.3→275.2 for 15-d-PGJ_2_-d_4_; and 327.0→226.2 for 15-HETE-d_8_. The levels of eicosanoids were expressed as pmol/mg protein, determined according to the Bradford method [65].

### 4.5. Statistical Analysis

The values are presented as average ± SD (for n = 15). The data were analyzed using one-way ANOVA with the Tukey’s post hoc test used for multiple comparisons to determine the significant differences between the groups. The statistical analysis for the two analyzed groups (granulocytes isolated from recovered and deceased COVID-19 patients, precented in Table 1) was performed using the unpaired *t*-test. A *p*-value < 0.05 was considered statistically significant. GraphPad Prism software, version 7.0, for Windows (GraphPad Software, San Diego, CA, USA) was used for all statistical analyses.

## 5. Conclusions

The obtained results indicate that the development of COVID-19 disturbs the metabolism of granulocytes, related to both the redox balance and the inflammatory processes. This is manifested by the decrease in antioxidant capacity at the level of both the cytosol and the mitochondria (SOD1/2) and an increased pro-inflammatory response (increased levels of NFkB(p52) and IL-6; decreased levels of IL-10) in the granulocytes of patients who died from the disease. These changes are accompanied by disorders at the level of enzymatic lipid metabolism, with increased generation of pro-inflammatory eicosanoids (TXB2 and PGE2) and decreased levels of anti-inflammatory eicosanoids (15d-PGJ2 and 5-HETE). As a consequence, this may cause dysregulation of the expression of G protein-coupled membrane receptors, which, by regulating the levels of ROS and TNFα, can modify the redox balance and inflammation, which may be directly related to the activation of immune cells.

Considering the relatively small number of patients included in this study, the results presented could be considered as preliminary, thus limiting the possibility of drawing specific conclusions as well as diagnostic and therapeutic suggestions. However, the possibility of validating the analyzed markers as predictors for the effects of treatment of patients with COVID-19 may be considered in the future. 

## Figures and Tables

**Table 1:** Comparison of selected laboratory data of patients with COVID-19 with respect to normal values and the outcome of the disease.

	Healthy People Normal Range n = 15 (7F + 8M)	COVID-19 Recovered n = 15 (7F + 8M)	COVID-19 Deceased n = 15 (7F + 8M)
Average age	55 (47–58)	57 (49–71)	66 (60–73)
Neutrophils (%)	40.0–72.0	78.52 ± 5.34	88.12 ± 4.87 *
Platelets (10^3^/μL)	150–400	287.34 ± 101.21	224.17 ± 59.46
Blood oxygen saturation (%)	>95%	91.32 ± 5.38	90.12 ± 5.42
Ferritin (μg/L)	11–336	901 ± 448	953 ± 451
PCT (ng/mL)	<0.1	0.39 ± 0.32	1.10 ± 0.71 *
LDH (U/L)	140–280	429 ± 127	347 ± 132
CRP (mg/L)	0.00–5.00	135.27 ± 71.24	181.75 ± 56.24
IL-6 (pg/mL)	0–43.5	84 ± 41	189 ± 96 *

*—significant (*p* < 0.05) difference with the values of recovered patients.

## Data Availability

The data presented in this study are contained within the article.
